# Biomechanical aspects of idiopathic scoliosis evolution

**DOI:** 10.1186/1748-7161-7-S1-O30

**Published:** 2012-01-27

**Authors:** AG Aulisa, V Guzzanti, C Perisano, RC Lavagna, G Scudieri, L Aulisa

**Affiliations:** 1 Children’s Hospital Bambino Gesù, Rome, Italy; 2 “A. Gemelli” Hospital, Università Cattolica del Sacro Cuore, Italy

## Background

In patients with idiopathic scoliosis, the interaction between biological and mechanical factors plays a central role in the evolution of deformities. According to the “vicious cycle model” of scoliosis evolution, the asymmetric load on the spine is the main factor driving the onset and development of deformities by altering the vertebral growth dynamics. Hence, once a critical asymmetric load has established, the progression of deformity is unavoidable, unless a compensatory force is applied to offset the biomechanical effects of growth [1,2].

Here, we present a case series of adolescents with idiopathic scoliosis, in whom a normal vertebral morphology was achieved before the end of growth, who withdrew from the orthotic treatment during the growing age and maintained the correction over a 5-year follow-up.

## Materials and methods

Fourty-six adolescents (40 girls and 6 boys) with idiopathic scoliosis treated with PASB or Lyon or Milwaukee brace, who achieved a complete curve and vertebral symmetry correction and withdrew from the treatment before the skeletal growth was complete. Participants presented with lumbar (n = 17), thoracolumbar (n = 26) or dorsal (n = 3) curve. The mean age at the beginning of treatment of 12.13 ± 2.16 years. All participants were prescribed with full-time bracing for an average of 53.35 ± 19.94 months. An early weaning was suggested, provided that a full-time bracing would be reinstituted if correction was lost. However, such a condition was not observed.

## Results

X-rays taken at the beginning of treatment showed a curve value of 23.93 ± 4.14° Cobb and Perdriolle value inferior to 15° Perdriolle. Radiologic examinations performed during the course of treatment evidenced a progressive reduction of vertebral rotation and lateral curvature, until the complete recovery of spinal geometry. At the end of treatment, all patients experienced a complete lateral curve correction. X-rays taken during the following 2 years showed a curve stabilization, with an average curve value of 4.80 ± 0.75° Cobb. Only 29 cases experienced a mild curve progression (7.62 ± 4.35 °Cobb).

## Conclusion

The mechanical component is the major force involved in curve evolution. The restoration of a normal vertebral geometry via conservative treatment stops the scoliosis evolution and results in a permanent correction of the curve.
